# An atlas of tsetse and animal African trypanosomiasis in Zimbabwe

**DOI:** 10.1186/s13071-020-04555-8

**Published:** 2021-01-14

**Authors:** William Shereni, Luis Neves, Rafael Argilés, Learnmore Nyakupinda, Giuliano Cecchi

**Affiliations:** 1 Division of Tsetse Control Services, Ministry of Lands, Agriculture, Water and Rural Resettlement, Harare, Zimbabwe; 2 Department of Veterinary Tropical Diseases, University of Pretoria, Pretoria, South Africa; 3 Centro de Biotecnlogia, Universidade Eduardo Mondlane, Maputo, Mozambique; 4 Joint Food and Agriculture Organization/International Atomic Energy Agency Programme, Vienna, Austria; 5 Animal Production and Health Division, Food and Agriculture Organization of the United Nations, Rome, Italy

**Keywords:** Atlas, *Glossina morsitans morsitans*, *Glosssina pallidipes*, Animal African trypanosomiasis, Epsilon trap, Database, Zimbabwe

## Abstract

**Background:**

In the 1980s and 1990s, great strides were taken towards the elimination of tsetse and animal African trypanosomiasis (AAT) in Zimbabwe. However, advances in recent years have been limited. Previously freed areas have been at risk of reinvasion, and the disease in tsetse-infested areas remains a constraint to food security. As part of ongoing control activities, monitoring of tsetse and AAT is performed regularly in the main areas at risk. However, a centralized digital archive is missing. To fill this gap, a spatially explicit, national-level database of tsetse and AAT (i.e. atlas) was established through systematic data collation, harmonization and geo-referencing for the period 2000–2019.

**Methods:**

The atlas covers an area of approximately 70,000 km^2^, located mostly in the at-risk areas in the north of the country. In the tsetse component, a total of 33,872 entomological records were assembled for 4894 distinct trap locations. For the AAT component, 82,051 samples (mainly dry blood smears from clinically suspicious animals) were collected at 280 diptanks and examined for trypanosomal infection by microscopy.

**Results:**

*Glossina pallidipes* (82.7% of the total catches) and *Glossina morsitans morsitans* (17.3%) were the two tsetse species recorded in the north and northwest parts of the country. No fly was captured in the northeast. The distribution of AAT follows broadly that of tsetse, although sporadic AAT cases were also reported from the northeast, apparently because of transboundary animal movement. Three trypanosome species were reported, namely *Trypanosoma brucei* (61.7% of recorded infections), *Trypanosoma congolense* (28.1%) and *Trypanosoma vivax* (10.2%). The respective prevalences, as estimated in sentinel herds by random sampling, were 2.22, 0.43 and 0.30%, respectively.

**Discussion:**

The patterns of tsetse and AAT distributions in Zimbabwe are shaped by a combination of bioclimatic factors, historical events such as the rinderpest epizootic at the turn of the twentieth century and extensive and sustained tsetse control that is aimed at progressively eliminating tsetse and trypanosomiasis from the entire country. The comprehensive dataset assembled in the atlas will improve the spatial targeting of surveillance and control activities. It will also represent a valuable tool for research, by enabling large-scale geo-spatial analyses.
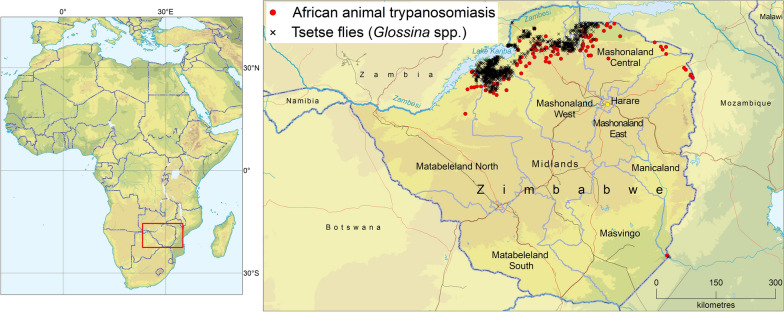

## Background

After foot and mouth disease (FMD) and tick-borne diseases (TBD), animal African trypanosomiasis (AAT) or Nangana [1] is the most economically important livestock disease in Zimbabwe. A closely related disease of humans, called human African trypanosomiasis (HAT) or sleeping sickness [2], also occurs sporadically in a few foci in the country. There is a constant need to monitor the distribution and prevalence of these diseases to guide evidence-based control.

Trypanosomes, the protozoan parasites that cause the diseases, are transmitted by tsetse flies (Genus: *Glossina*) [3]. AAT is caused mainly by three species of trypanosome, namely *Trypanosoma vivax*, *T. congolense* and *T. brucei*, whereas HAT is caused by two sub-species of *T. brucei*, i.e. *T. b. rhodesiense* in southern and eastern Africa and *T. b. gambiense* in western and central Africa [2]. While HAT is in the process of being eliminated from the continent [4, 5], progress in AAT control is much slower [6]. Nagana still ravages livestock in sub-Saharan Africa, and the combination of direct and indirect impacts is estimated to be in excess of 4.75 billion US dollars every year [7–9].

The tsetse-infested area in Zimbabwe is currently estimated at 30,000 km^2^, which corresponds to 17% of the 180,000 km^2^ ecologically suitable area originally infested by tsetse [10]. In particular, tsetse distribution in Zimbabwe has always been restricted to the north, northwest, northeast and southeast of the country with the central highveld being ecologically unsuitable for the fly.

Currently, only two species of tsetse, i.e. *Glossina morsitans morsitans* and *G. pallidipes*, are reported to occur in Zimbabwe [11]. The distribution of AAT in Zimbabwe follows closely the distribution of tsetse, although this is not always the case in other affected countries. For example, in the Sahel and the Sudanian savannahs of northern Africa, mechanical transmission by other vectors (e.g. tabanids and Stomoxys) as well as animal movement (e.g. pastoralism) brings the disease far beyond the tsetse belts [12, 13]. In Zimbabwe, however, Boyt et al. [14] found no evidence of mechanical transmission in the southeast of the country.

The foci of *rhodesiense* HAT in Zimbabwe are in the Hurungwe and Kariba Districts in the north of the country [5, 11, 15, 16]. The cases include a number of foreign travelers diagnosed outside the country [16].

The present distribution of tsetse and AAT in Zimbabwe is the result of a long and complex interplay of land cover, climate, epidemiological factors and control measures. This complex history includes the rinderpest epizootic in the late nineteenth century, major tsetse control operations implemented in the 1960s and 1970s, and then again in the 1980s and 1990s following the war of independence, which led to the creation of large tsetse-free areas [17, 18]. Since 2000, however, funding for tsetse and AAT control has decreased, so large-scale operations were scaled down. The emphasis shifted to the protection of previously cleared areas and the consolidation of past gains.

The control of tsetse and AAT in Zimbabwe is the responsibility of the Division of Tsetse Control Services (TCD) and the Division of Veterinary Field Services (DVFS), respectively. Both units are part of the Department of Veterinary Services (DVS), with the TCD being responsible for the management of tsetse and the compilation of AAT data and the DVFS in charge of disease management, in particular the operation of diptanks and spray races.

The total staff establishment of the TCD is over 500, comprising 55 based at the headquarters (HQ) in Harare and approximately 50 in each of its nine field station. A Geographic Information System (GIS) and data management unit are based in Harare.

The last national map of AAT distribution in Zimbabwe was published in 1999 by Van Den Bossche and Vale [19]. A substantial amount of monitoring data has been collected over the years, and procedures for data transmission and management exist. However, a unified, centralized and regularly updated database was never developed, thus hampering evidence-based decision-making. Against this backdrop, the FAO continental atlas of tsetse and AAT [13, 20, 21] provided the blueprint to develop a national atlas in Zimbabwe and in other countries [12, 22, 23]. The development of national atlases was also spurred by the need to inform the progressive control pathway (PCP) for AAT [6]. PCPs are risk-based, stepwise strategic approaches to plan and monitor the reduction or elimination of diseases. Epidemiological mapping hinging on geo-spatial data management systems is crucial for a successful roll-out of PCPs at the national level. Zimbabwe is one of the first countries to have initiated the adoption of this approach for tsetse and AAT control, and the development of the national atlas is its first major initiative in the PCP framework.

## Methods

The national atlas initiative was launched in 2016, with a focus on the systematic collation and harmonization of tsetse and AAT data collected in the field from the year 2000 onwards. At inception, most of the necessary data were already available at the HQ, through the monthly reporting from field stations. However, a number of gaps were identified, and requests for the missing data were sent to field stations.

The vast majority of the atlas data originate from regular monitoring by the TCD and have not been published before.

### Input data

#### Tsetse data

The tsetse monitoring system in Zimbabwe hinges on odor-baited epsilon traps, including fixed sites that have been operated for > 5 years, as well as other sites employed for spot checks lasting from a few days to a few months. Species and sex identification is carried out in the field by trained technicians. The trap used is the Epsilon [24], usually supplied with odor attractants comprising 3-n propyl phenol, 1-octen-3-ol and 4-methyl phenol, in a 1:4:8 ratio, and a ketone, either acetone or butanone. All odors were dispensed as described by Torr [25]. Traps were deployed in representative vegetation types in more open and visible sites according to the siting principles of Vale [26].

The fixed monitoring traps were serviced daily or monthly for research purposes at Rekomechi field station and at least once every 3 months (average 2 days) in operational areas. Monitoring sites were located both inside the known area of tsetse infestation and in the adjacent areas expected to be free of tsetse. The latter areas are normally protected against reinvasion by barriers of insecticide-treated targets (ITT) [27, 28] and/or insecticide-treated cattle (ITC) [29].

In addition to the fixed monitoring sites, tsetse distribution and apparent densities were also monitored on a spot basis in semi-permanent sites, with the aim to complement and refine information coming from fixed sites. Some spot checks were in supposedly tsetse-free areas where cases of AAT were reported, with a view to confirming the possibility of local transmission.

For each monitoring event the tsetse apparent density is calculated as the total catch from a trap divided by the number of days [flies/trap/day].

#### Animal African trypanosomiasis

A nation-wide network of diptanks and spray races exists in Zimbabwe for the control of both ticks and, where present, tsetse. From the sites in areas affected by, or at risk of, AAT, blood samples for parasitological monitoring were collected by the DVFS and sent to the TCD laboratory in Harare for analysis. Microscopic examination of dry thin smears [30] is the most commonly used diagnostic technique. A 40× or 50× magnification is used to determine if samples are positive, and 100× is used to identify the species of trypanosome in positive samples. Morphological features such as the shape, size, the characteristics of the undulating membrane and the flagellum, and the position of the kinetoplast and the nucleus are used in determining the species of trypanosome.

Blood samples are normally collected from clinically suspicious animals. This approach has its drawbacks. For example, when cattle are treated with drugs by farmers parasitemia levels are lowered, thus reducing the likelihood of trypanosome detection. Prevalence data presented in this paper are based on the examination of infections in sentinel herds managed by the TCD, where blood samples were collected from the entire herd. Occasionally, wet smears were collected and directly analyzed in the field to administer treatment on the spot. Overall, nine sentinel herds are managed by the TCD, one in each field station, and they are strategically deployed in different locations to monitor the AAT situation in the affected areas and as an early-warning mechanism for possible disease resurgence in AAT-free areas.

### Structure of the atlas

The atlas of tsetse and AAT for Zimbabwe comprises two major components, i.e. the data repository and the database.

#### Data repository

The data repository includes a digital version of all input files that were used to create the database.

For the tsetse component, the original source of information is the recording sheets that were compiled by field technicians. The original sheets were filed in the field stations and hard copies sent to HQ for compilation and analysis. Most data entry into digital spreadsheets is carried out at HQ.

For the AAT component, the main source of information is available in hard copy at the TCD laboratory, and it includes the results of microscopic examination of the dry thin blood smears from individual animals. To develop the atlas, this information was entered into spreadsheets and aggregated by diptank and by survey period.

#### The database

Additional file 1: Text S1 describes in detail the structure of the database. The component dealing with tsetse occurrence is recorded at the level of individual traps and includes the following information: (1) survey period/dates, (2) type of trap (epsilon in all cases), (3) odor attractant used, (4) number of trappings in days, (5) number of flies captured (by sex and species) and (6) apparent density (AD), i.e. flies/trap/day.

Information for the AAT component includes: (1) the survey period, (2) diagnostic method, (3) number of animals tested, (4) animal species and sex and (5) husbandry system. Note is taken of the type of sampling (i.e. clinically suspicious animals, random sampling or systematic testing of an entire sentinel herd). Information on the use of trypanocidal drugs prior to the survey is also recorded. Trypanosomes were identified only to species level since the diagnostic techniques did not enable sub-species and sub-groups/types to be distinguished. Mixed infections were noted.

For mapping purposes, geographical coordinates were recorded as latitude and longitude on the WGS84 datum. The names of the province, district, field station and location of the spray race or diptank were also recorded.

The geo-referencing of the tsetse data is at the level of individual traps. Before 2009, mapping of the trap position relied on topographical maps and traditional cartographic techniques. Thereafter, the Geographic Positioning System (GPS) replaced traditional cartography.

Data on the AAT component of the atlas were mapped at the level of the diptank or spray race where samples were collected or the site where the sentinel herds were located. All sites/locations were geo-referenced with GPS.

### Atlas development process

Although there is regular reporting of tsetse and AAT data from field stations to the Harare HQ, some of the data there were destroyed by fire. Therefore, for the development of the atlas, templates were sent out to the field stations, and all the data available for the study period were transmitted again to the HQ. Thereafter, data harmonization was carried out, for example to ensure consistency in the format of geographic coordinates and dates. Data verification as well as quality control to remove data entry errors was a time-consuming step.

## Results

**Fig. 1:**
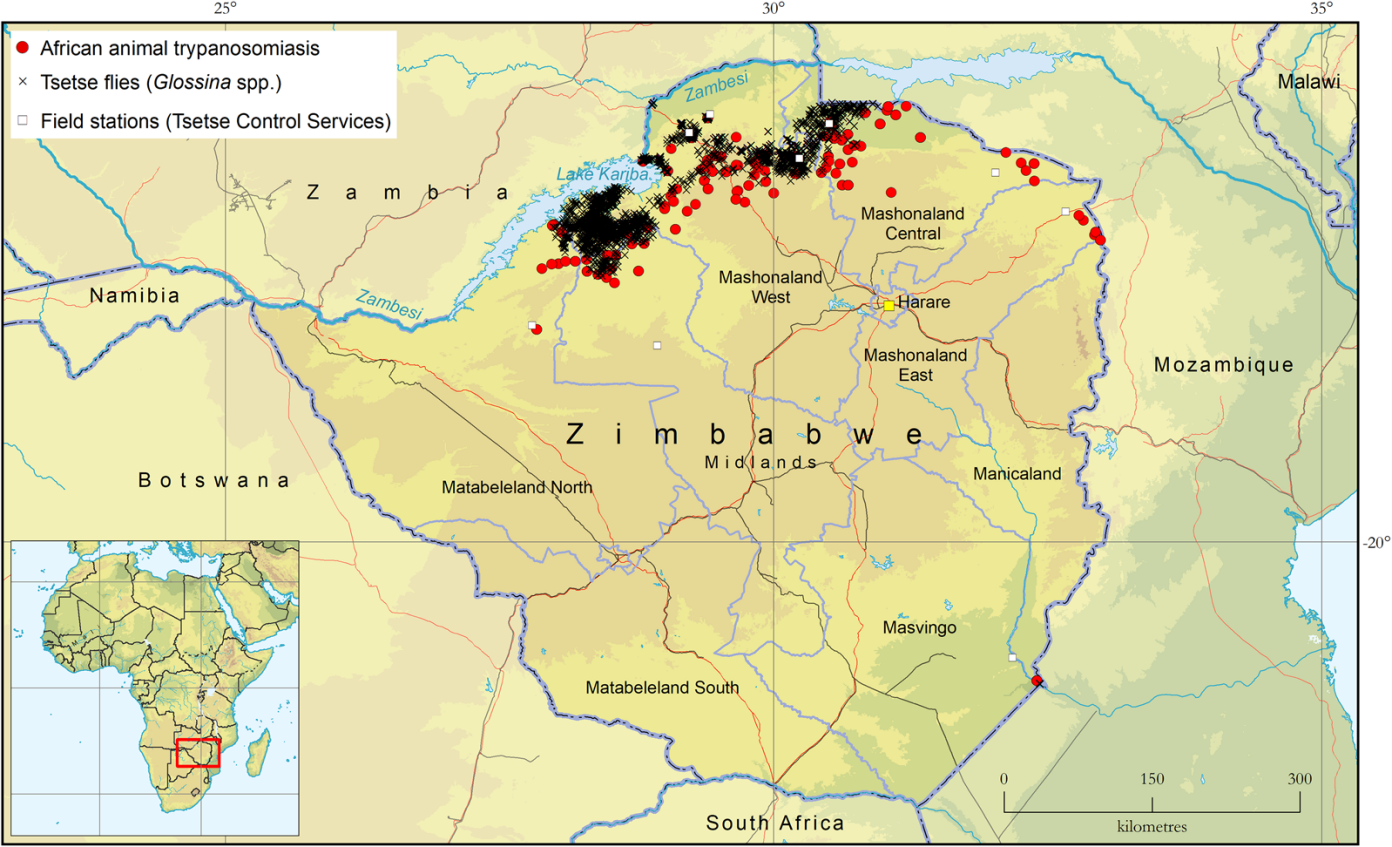
Occurrence of tsetse and animal African trypanosomiasis in Zimbabwe: 2000–2019

The results of the atlas are summarized in Fig. 1. This shows the reported occurrence of AAT and tsetse flies in Zimbabwe in the period 2000–2019

### Tsetse distribution

The atlas of tsetse presently comprises 4894 distinct trapping sites, which were monitored from a minimum of 2 days to a maximum of 3921 days (average 166 days). A total of 33,872 entomological records/trapping events were included in the database.

Over 99% of the entomological records include geographic coordinates. All records include the month and year of trapping, and approximately 51% also include the exact days of deployment and monitoring. The exact trapping duration is available for 21,180 records (63%), which was used to calculate apparent densities (flies/trap/day). For the 63% of records that include information on the duration of trapping, the cumulative trapping intensity is in excess of 570,000 trap days. This corresponds to an estimated overall trapping intensity close to 1 million trap days, if records without trapping durations are considered.

**Fig. 2:**
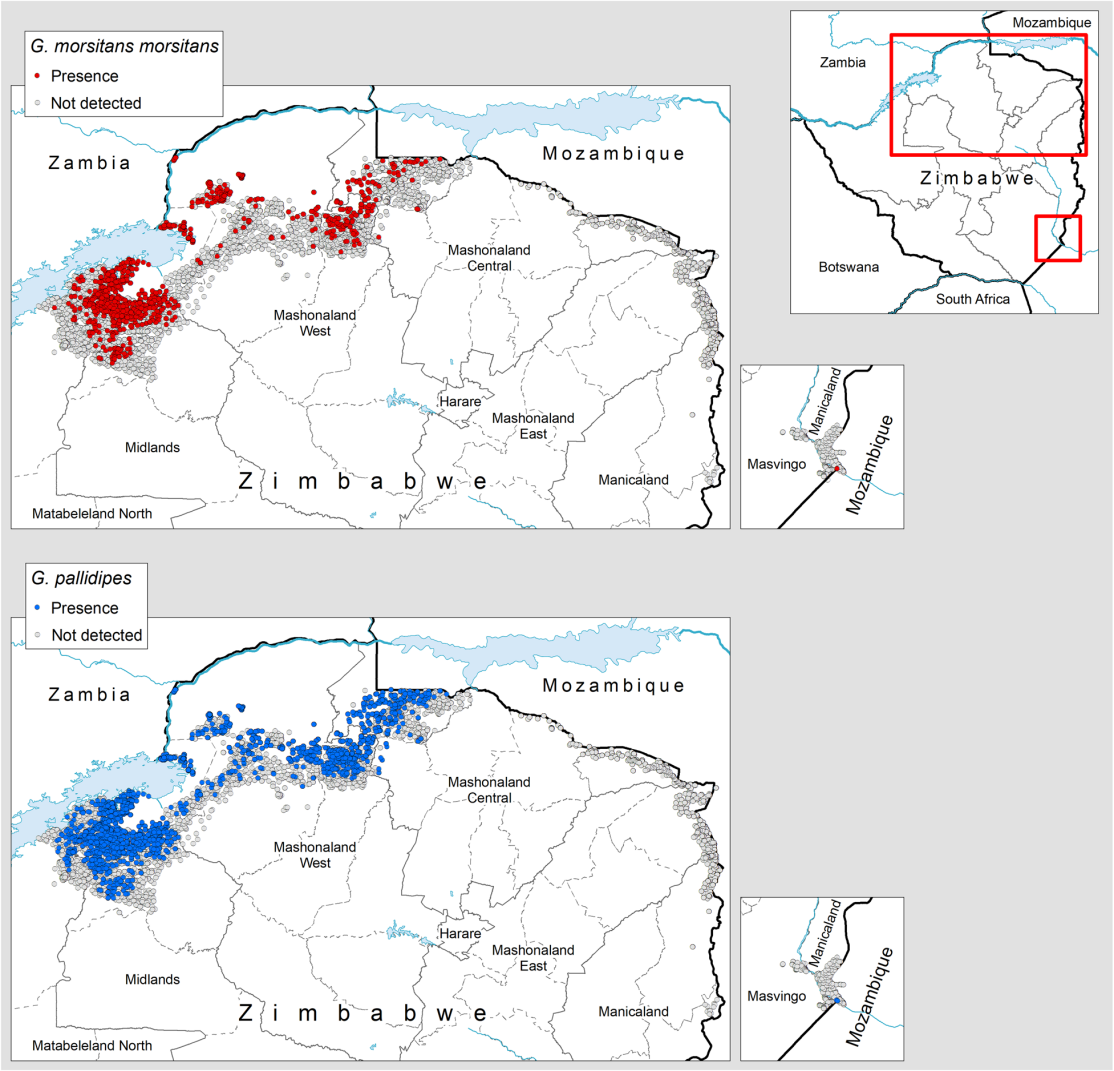
Presence (colored circles) and absence (surveyed but not detected, grey circles) of *G. m. morsitans* and *G. pallidipes* in Zimbabwe: 2000–2019

The total number of tsetse caught in the study period was 995,749, comprising 823,807 *G. pallidipes* and 171,942 *G. m. morsitans*. The mean apparent densities were 0.91 and 0.13 flies/trap/day, respectively. The presence/absence of capture by species is shown in Fig. 2. No G. *brevipalpis* and *G. austeni* were reported, although these species were occasionally reported until the 1990s along the border with Mozambique [31].

Tsetse are confirmed to be present in the north and northwest parts of the country. By contrast, substantial trapping efforts in the northeast along the border with Mozambique (i.e. > 44,000 trap days in > 160 different trapping locations covering the entire period 2000–2019) resulted in no catches. Limited monitoring in the SE detected flies in only one location, where the last tsetse was caught in 2004.

### Animal African trypanosomiasis distribution

**Fig. 3:**
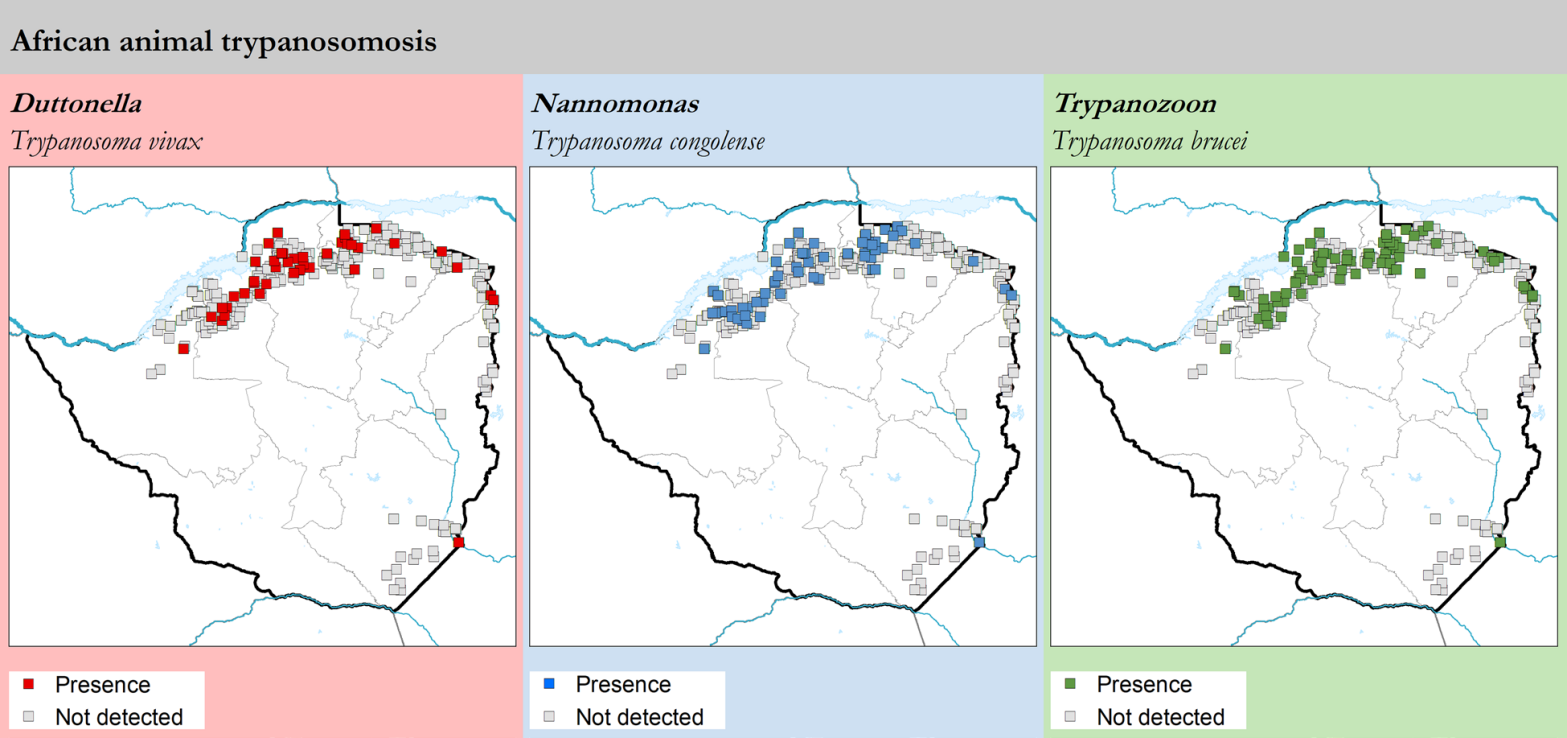
Presence (colored squares) or absence (surveyed but not detected, gray squares) of *T. vivax, T. congolense* and *T. brucei* in blood smears collected in Zimbabwe in 2000–2019

The AAT component of the atlas comprises 4178 parasitological records collected from 280 sites. The overall sampling intensity was 82,080 animals, with the detection of 1260 *T. brucei* infections, 574 *T. congolense* and 207 *T. vivax*. The reported geographic distribution of AAT at the national level is shown in Fig. 1 while the maps of presence and absence by trypanosome species are shown in Fig. 3.

The distribution of AAT in Zimbabwe appears to be closely related to the tsetse distribution, in particular in the tsetse-affected areas in the north and northwest. The sporadic positive cases of AAT detected in the northeast, along the border with Mozambique, seem to be imported infections, since follow-up investigations by DVFS and TCD revealed transboundary animal movement and yielded no new AAT cases or tsetse catches. In the southeast of the country AAT cases were detected in a sentinel herd, the last case having been reported in 2005.

Trypanosome prevalences in sentinel herds were 2.22, 0.43 and 0.30% for *T. brucei, T. congolense* and *T. vivax*, respectively. Despite these differences in prevalence, no major difference in the geographic distribution of trypanosome species is apparent.

### Time series and changes in the distribution of tsetse and trypanosomiasis

**Fig. 4:**
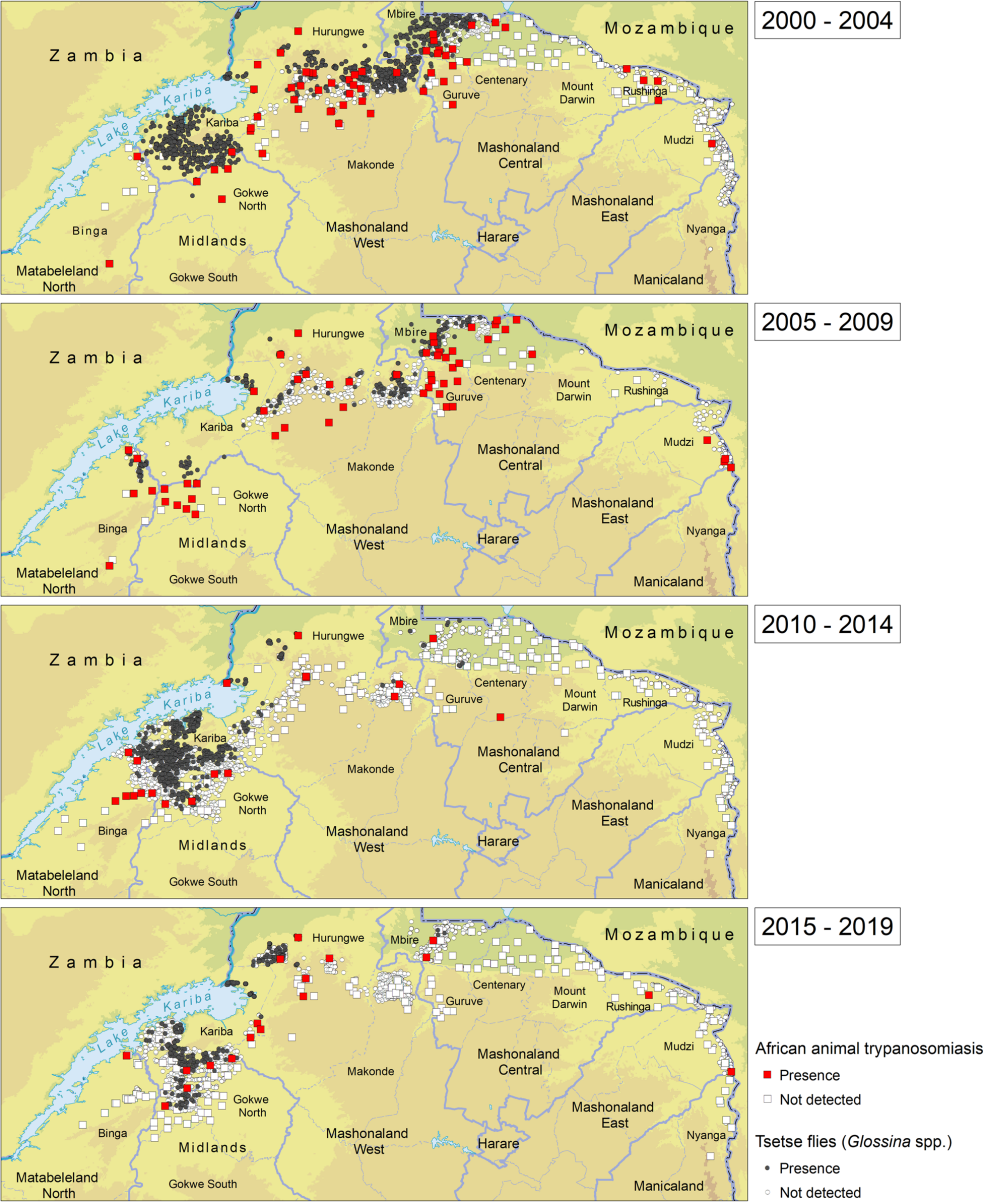
Time series of tsetse and AAT absence/presence: 2000–2019

Figure 4 shows the evolution of the tsetse and AAT distribution in northern Zimbabwe during the 20-year study period in four time windows. Despite an uneven coverage of surveillance activities, a few major patterns can be observed. In particular, a reduction in tsetse distribution and a decline in AAT occurrence were observed. The decline of tsetse populations is particularly evident in the central part of northern Zimbabwe (i.e. Mbire district and the northern part of Makonde district), but also visible to a lesser extent in areas to the West (i.e. Hurungwe and Kariba districts). In the eastern part, no fly was captured during the whole study period, although a reduction in the geographical coverage of surveillance is visible. Similar albeit less stark partners can be observed for the occurrence of AAT in space and time.

## Discussion

The goal of assembling, harmonizing and geo-referencing tsetse and AAT data collected by the TCD and DVFS for the period 2000–2019 has been achieved. This national atlas for Zimbabwe follows the ones for Sudan [12], Mali [22] and Kenya [23], but vastly exceeds them in terms of the number of data points. In particular, the overall trapping effort in Zimbabwe is approximately 1 million trap days, compared to < 20,000 trap days in other countries.

The atlas shows that tsetse and AAT in Zimbabwe occur in the north and northwest. The absence of flies in the northeast, an area well covered by the atlas, is mainly explained by extensive tsetse control interventions implemented before 2000, involving ITT, ITC, ground spraying and sequential aerial spraying, but also by the sustained use of ITT and ITC in the following 2 decades.

This first version of the atlas enables gaps in the geographic coverage of surveillance to be identified. In particular, the data assembled in the atlas cover an area of approximately 70,000 km^2^ (i.e. < 20% of the country surface area). Hardly any recent information is available on the vast areas of the country that are assumed to be free of tsetse and AAT. Also, data are lacking from parts of the Mana Pools National Park in the Zambezi Valley, where tsetse are known to be present.

The atlas will be regularly enhanced, updated and, to the extent possible, expanded to include older datasets (i.e. before the year 2000). Data collected for research and published in the scientific literature also need to be systematically included. Plans are also in place to migrate the database from its present format (i.e. two plain Microsoft Excel files, one for AAT and one for tsetse) to more advanced database management systems (e.g. Microsoft Access).

Developing the atlas also allowed strengthening and streamlining data reporting from the field to the central level by harmonizing the reporting formats. In addition to the maps of presence and absence presented in the results section, the atlas allows maps of the observed tsetse apparent density and AAT prevalence to be generated.

Ideally, a map of the apparent density of tsetse would have been presented, but this was hindered by the lack of standard procedures in the trapping system. For example, there were variations in the odors employed, the sorts of collector (cage) employed on top of the traps, the types of cloth used for the body of the traps and the frequency of trap emptying. We are currently investigating the effects of these variations, with a view to correcting the observed catches and allowing a comparison of apparent densities. For now, our focus was on the presence/absence of information presented. The latter was also affected by the lack of standard procedures in the trapping system, but to a lesser degree, in that when the presence of a certain tsetse species is confirmed in a given location, the information can be used with less regard to the specificities of the trapping system.

As to the AAT component of the atlas, its geographical coverage is adequate for decision-making. However, in the context of the PCP for AAT and the OIE criteria for declaring freedom from the disease, it will be necessary to produce data to confirm the presumed AAT-free status of many areas. The fact that the AAT distribution appears to follow the tsetse distribution closely seems to indicate that, in Zimbabwe, the capacity of mechanical vectors and animal movement to spread the disease in tsetse-free areas may be very limited. At the same time, the transboundary nature of the disease is apparent in the northeast where, due to cross-border animal movement, sporadic AAT cases were detected in an area that is believed to be free of tsetse.

AAT survey results in the atlas (Fig. 4) are similar to those in a survey conducted in 1997 by Torr [32] as both were based on microscopic examination of samples. Overall, more cases were detected in the survey by Van den Bossche [19], which spanned from January 1998 to September 1999, as both microscopy and the enzyme-linked immunosorbent assay (ELISA) were used. Parasitological surveys gave comparable results except in the northern region where more cases were detected. The reduction of cases in the north could be attributed to the increased resettlement of people and the related extensive deforestation for agricultural activities and for curing tobacco. Furthermore, the differences could also be due to reduced tsetse invasion and cattle dipping in the northeast and southeast, target operations, ground spraying and cattle dipping in the northwest and target operations and cattle dipping in the north. In most areas surveyed, the true AAT prevalence situation was distorted by the increased informal and unreported usage of curative drugs by farmers, a situation that reduces parasitemia levels and therefore chances of detecting the disease by microscopy.

One of the main weaknesses of the AAT component of the atlas is the scarcity of randomized sampling in AAT surveys, which limits the ability to estimate disease prevalence. Another weakness is related to the generally low sensitivity of the blood-smear method of diagnosis. Furthermore, reduced parasitemia, due to undeclared treatment of cattle with trypanocidal drugs by farmers, can reduce the chances of detection of trypanosomal infections through traditional microscopy. Recently, the sensitivity of the AAT diagnostic protocols has been improved by establishing a polymerase chain reaction (PCR) unit to enable identification to the sub-species and subgroup level. Data originating from molecular analysis will be included in future versions of the atlas.

A broader utilization of PCR diagnosis could also help confirm our finding of the preponderance of *T. brucei* infections in Zimbabwe, a finding that is fairly consistent with some previous studies [33, 34] but not with others [35, 36].

The data assembled in the atlas will be useful for a range of analyses and modeling [37, 38], for example, modeling effects of climate, human settlement and control interventions on tsetse and to inform the Progressive Control Pathway (PCP) for AAT.

## Conclusion

The national atlas of tsetse and AAT represents a key tool for data management and can be used to inform tactical and strategic decision-making in tsetse and AAT control. Establishing an atlas has enabled existing data collection and reporting procedures to be refined and their accuracy and robustness enhanced. The atlas is useful in delineating areas currently infested with tsetse and in monitoring changes in tsetse distribution, thereby providing a basis for identifying priority areas for control interventions.

## Supplementary Information

**Additional file 1: Text S1.** Structure of the database on tsetse and animal African trypanosomiasis in Zimbabwe.

## Data Availability

Relevant data are within the paper and its Supporting Information files. The bulk of the data on the tsetse and AAT occurrence in Zimbabwe is the property of the Government of Zimbabwe, Division of Tsetse Control Services, and data can be requested from: Director, Division of Tsetse Control Services, P.O Box: CY52, Causeway, Harare, Zimbabwe, Phone: +263242707365 / +263772545992, E-mail address: shereni2005@yahoo.com.

## Abbreviations

AAT: Animal African trypanosomiasis
HAT: Human African trypanosomiasis
PCP: Progressive control pathway
TCD: Division of Tsetse Control Services
DVS: Department of Veterinary Services
DVFS: Division of Veterinary Field Services
HQ: Headquarters
GIS: Geographic Information System
GPS: Global Positioning System
FAO: Food and Agriculture Organization of the United Nations
ITC: Insecticide-treated cattle
ITT: Insecticide-treated targets
