# Supplementary material for: An atlas of tsetse and animal African trypanosomiasis in Zimbabwe
Source: Parasit Vectors. 2021 Jan 14;14:50. doi: 10.1186/s13071-020-04555-8 (PMC7807824; doi:10.1186/s13071-020-04555-8)
Supplement: Supplementary file 1 — Additional file 1: Text S1. Structure of the database on tsetse and animal African trypanosomiasis in Zimbabwe. [file 13071_2020_4555_MOESM1_ESM.docx]

# Structure of the database on tsetse and animal African trypanosomiasis in Zimbabwe

# Protocol of data abstraction

# Occurrence and distribution of tsetse

The protocol describes how data on the occurrence and distribution of tsetse, and occurrence and prevalence of African animal trypanosomiasis (AAT), are assembled into a geospatial database within the framework of “the Atlas of tsetse and AAT” initiative. Data are extracted from data sheets and internal reports, captured into excel spreadsheets and stored in a central data repository. Extracted data are assembled in a geographic form using excel. Information items included this table are described below.

## Geographical and epidemiological data

The tsetse Atlas table contains detailed information on the geographical scope of study and it includes geo-positioning information. We define as ‘location’ the geographic location where the study was carried out, and it constitutes the basic unit for mapping. All sites are represented as points in the Atlas of tsetse. The following fields are recorded in the table ‘Atlas_tsetse’:

1. SOURCE. The source containing the site and entomological data (it will in future be contained in a table ‘Sources’).
2. STATION. Name of the station responsible for data collection in the study area.
3. YEAR. The year in which data was compiled.
4. MONTH. The month in which data was compiled.
5. LOCATION. Name of the site where the study was conducted. As a rule, the name as reported in the data sheets/ report is recorded.
6. PROVINCE. Name of the Province where the site is located (ADMIN 1 – first subnational administrative unit where the site is located.
7. DISTRICT. Name of the District where the site is located (ADMIN 2 – second subnational administrative unit where the site is located.
8. TRAP_CODE. Unique identifier assigned to each trap.
9. LONGITUDE. Longitude of the study site in decimal degrees (Datum: WGS84).
10. LATITUDE. Latitude of the study site in decimal degrees (Datum: WGS84).
11. VEGETATION. Type of vegetation around the site.
12. LANDUSE. The purpose for which the land on which the site is located is used for e.g Safari Area.
13. DAY_ST. Starting day of the survey.
14. MONTH_ST. Starting month of the survey.
15. YEAR_ST. Starting year of the survey.
16. DATE_ST. Full starting date of the survey
17. DAY_EN. Ending day of the survey.
18. MONTH_EN. Ending month of the survey.
19. YEAR_EN. Ending year of the survey.
20. DATE_EN. Full ending date of the survey.
21. TRAP_DAYS_CALC. Number of days of trapping (Where dates are available “DATE_EN” subtract “DATE_ST”).
22. TRAP_DAYS. Number of days of trapping (numeric value corresponding to TRAP_DAYS_CALC in cases where dates are available, and where dates are unavailable number of days if known).
23. MGM. Total number of male *Glossina morsitans* caught in a trap.
24. FGM. Total number of female *Glossina morsitans* caught in a trap.
25. GM_TOT. Total number of *Glossina morsitans* (“MGM” + “FGM”) caught in a trap.
26. MGP. Total number of male *Glossina pallidipes* caught in a trap.
27. FGP. Total number of female *Glossina pallidipes* caught in a trap.
28. GP_TOT. Total number of *Glossina pallidipes* (“MGP” + “FGP”) caught in a trap.
29. TOTAL. Total number of all flies (“GM_TOT” + “GP_TOT”) caught in a trap
30. AD_GM. Apparent density of *Glossina morsitans* (“GM_TOT” divided by “TRAP_DAYS”)
31. AD_GP. Apparent density of *Glossina pallidipes* (“GP_TOT” divided by “TRAP_DAYS”)
32. AD_TOT. Apparent density of all flies (“TOTAL” divided by “TRAP_DAYS”)
33. TABANIDS. Number of tabanids caught in a trap
34. STOMOXYS. Number of stomoxys caught in a trap
35. HIPP. Number of Hippoboscidae caught in a trap
36. ATTRACTANT. Name of attractants used with the trap
37. NOTES. Includes all important additional information as reported in the source.

# Animal African trypanosomiasis

## Geographical and occurrence data

The trypanosomiasis Atlas table contains detailed information on the geographical scope of study and it includes geo-positioning information. We define as ‘location’ the geographic location where the samples were collected, and it constitutes the basic unit for mapping. All sampling sites are represented as points in the Atlas of trypanosomiasis. The following fields are recorded in the table ‘Atlas_AAT’:

1. STATION. Name of the station operating in the area where sampling sites are located.
2. DAY_ST. Starting day of the survey.
3. MONTH_ST. Starting month of the survey.
4. YEAR_ST. Starting year of the survey.
5. DATE_ST. Full starting date of the survey.
6. PROVINCE. Name of the Province where the site is located (ADMIN 1 – first subnational administrative unit where the site is located.
7. DISTRICT. Name of the District where the site is located (ADMIN 2 – second subnational administrative unit where the site is located.
8. RACE_DIPTANK_NAME. Name of the sampling site which usually is a dip tank or race and in our case could be sentinel herd location.
9. LONGITUDE. Longitude of the sampling site in decimal degrees (Datum: WGS84).
10. LATITUDE. Latitude of the sampling site in decimal degrees (Datum: WGS84).
11. AREA: Surface area of the survey (area of applicability for the recorded AAT data)
12. CENSUS: Total number of animals expected at sampling site.
13. SAMPLE_SIZE: Actual number of animals sampled.
14. DIAGNOSTIC­_METHOD. Method used to collect and examine samples for trypanosomiasis.
15. CHEMOTHERAPY: Were therapeutic and/or prophylactic antitrypanosomal drugs used (Yes/No).
16. DRUGS_USED: Actual therapeutic and/or prophylactic antitrypanosomal drugs used to treat the animals.
17. TSETSE_CONTROL_INTERVENTIONS: it reports what interventions against tsetse were ongoing in the study area at the time of the survey, or in the recent past prior to the survey.
18. SAMPLING_METHOD: it describes whether a random approach was used (e.g. in a study designed to assess the general epidemiological situation in an area), or a purposeful one (e.g. where specific villages/areas/herds/animals are investigated because of some peculiar features of theirs)
19. RANDOM. Explains whether sampling was random or not (Yes/No)
20. SPECIES: species of animal (e.g. Cattle, goats, sheep, pigs, etc...).
21. BREED: animal breed. Because of the difficulties of harmonizing the descriptions and denominations of animal breeds, the sections of the paper referring to breeds are reported verbatim in this field.
22. AGE: age of animals. Because of the difficulties of harmonizing the descriptions of animal ages, the sections of the paper referring to ages are reported verbatim in this field.
23. SEX: sex of animals.
24. HUSBANDRY: Animal husbandry system. Because of the difficulties of harmonizing the descriptions of husbandry system, the sections of the paper referring to husbandry systems are reported verbatim in this field. Attention is given to whether animals are kept in a prevalently sedentary system, or semi-pastoral/transhumant one, or pastoral. More in general, all aspects that have implications in terms of exposure to infection are noted, including grazing versus zero-grazing, extensive versus intensive, commercial versus small-hold enterprises, etc.
25. HERD_TYPE: Relates to whether the animals belong to the communities or Tsetse Control Services (Sentinel).
26. INFECTION_STATUS. Describes whether animal is positive or negative.
27. NUM_INFECTED. Number of animals infected with trypanosomes.

*Infections with individual trypanosome species/subspecies/subgroups*

1. Tc: Number of animals positive to the test for *T. congolense*.
2. Tb: Number of animals positive to the test for *T. brucei* s.l.
3. Tv: Number of animals positive to the test for *Trypanosoma vivax*.
4. Tv_Tb. Number of animals positive with mixed infections of *Trypanosoma vivax* and *T. brucei* s.l.
5. Tc_Tb. Number of animals positive with mixed infections of *Trypanosoma congolense* and *T. brucei* s.l.
6. Tv_Tc. Number of animals positive with mixed infections of *Trypanosoma vivax* and *T. congolense*.

Note that animals diagnosed with a mixed infection (infections with more than one species/sub-species/subgroup of trypanosomes) are included in the counts for the respective infections with individual species.

1. NOTES: Includes all important additional information as reported in the source.
